# Integrated framework for quantitative T2-weighted MRI analysis following prostate cancer radiotherapy

**DOI:** 10.1016/j.phro.2024.100660

**Published:** 2024-10-24

**Authors:** Evangelia I. Zacharaki, Adrian L. Breto, Ahmad Algohary, Veronica Wallaengen, Sandra M. Gaston, Sanoj Punnen, Patricia Castillo, Pradip M. Pattany, Oleksandr N. Kryvenko, Benjamin Spieler, John C. Ford, Matthew C. Abramowitz, Alan Dal Pra, Alan Pollack, Radka Stoyanova

**Affiliations:** a Department of Radiation Oncology, University of Miami Miller School of Medicine, Miami, FL, USA; b Desai Sethi Urology Institute, University of Miami Miller School of Medicine, Miami, FL, USA; c Department of Pathology and Laboratory Medicine, University of Miami Miller School of Medicine, Miami, FL, USA; d Department of Radiology, University of Miami Miller School of Medicine, Miami, FL, USA

**Keywords:** Prostate cancer, T2-weighted MRI, Radiotherapy, Image registration, Intensity normalization, Harmonization, Quantitative imaging

## Abstract

**Purpose:**

The aim of this study is to develop a framework for quantitative analysis of longitudinal T2-weighted MRIs (T2w) following radiotherapy (RT) for prostate cancer.

**Materials and methods:**

The developed methodology includes: *(i)* deformable image registration of longitudinal series to pre-RT T2w for automated detection of prostate, peripheral zone (PZ), and gross tumor volume (GTV); and *(ii)* T2w signal-intensity harmonization based on three reference tissues. The *RE*gistration and *HARM*onization (*REHARM*) framework was applied on T2w acquired in a clinical trial consisting of two pre-RT and three post-RT MRI exams. Image registration was assessed by the DICE coefficient between automatic and manual contours, and intensity normalization via inter-patient histogram intersection. Longitudinal consistency was evaluated by the repeatability coefficient and Pearson correlation (*r*) between the two T2w exams before RT.

**Results:**

T2w from 107 MRI exams (23 patients) were utilized. Following *REHARM*, the histogram intersections for prostate, PZ and GTV increased from median = 0.43/0.16/0.13 to 0.66/0.44/0.46. The repeatability in T2w intensity estimation was better for the automatic than the manual contours for all three regions of interest (*r* = 0.9, *p* < 0.0001, for GTV). The changes in the tissues’ T2w values pre- and post-RT became significant, indicating the measurable quantitative signal related to radiation.

**Conclusions:**

The developed methodology allows to automate longitudinal analysis reducing data acquisition-related variation and improving consistency. The quantitative characterization of RT-induced changes in T2w will lead to new understanding of radiation effects enabling prediction modeling of RT response.

## 1 Introduction

Prostate cancer has a long natural history and local persistence after primary treatment can remain clinically undetectable for over 10 years. The role of quantitative imaging as early surrogate biomarker of RT response, especially from longitudinal studies involving multiparametric MRI (mpMRI) before and after radiation treatment (RT), is crucial for assessing treatment efficacy, guiding therapeutic adjustments, and predicting patient outcomes more accurately [1]. Longitudinal analysis of mpMRI series is challenging because of the need for precise registration of the data obtained at several time points. The prostate volume changes following RT and the magnet/sequence causes signal intensity variability, particularly in the setting of longitudinal data acquisition where multiple magnets and sequences are potentially used.

The RT response has been previously characterized by extracting quantitative features from the functional sequences of mpMRI, Dynamic Contrast Enhanced (DCE-) MRI and Apparent Diffusion Coefficient (ADC) maps, related to vascular perfusion/permeability and tissue water diffusion restriction and cellularity [2], [3], [4], [5]. The intrinsic tissue T2 relaxation times provide excellent contrast between the tissues and are potentially suitable to characterize the RT-induced changes [6], [7]. However, the standard of care clinical protocols for mpMRI include only T2-weighted MRI (T2w) sequence and do not include sequences for T2 estimation. While T2w is the most commonly used mpMRI sequence in the prostate, RT-induced tissue changes in prostate cancer and normal prostate tissue identified in serial T2ws before and after RT have not been thoroughly investigated.

In this study an automated image analysis framework was developed and applied to localize and characterize longitudinal changes in the prostate, peripheral zone (PZ) and Gross Tumor Volumes (GTVs) following RT. Longitudinal consistency in assessment of T2w was achieved through automated mapping of the regions of interest (ROIs) using deformable image registration, followed by a T2w intensity normalization technique. The *RE*gistration and *HARM*onization (*REHARM*) framework was assessed on longitudinal MRI data acquired pre-RT (two timepoints) and post-RT (three timepoints) from patients enrolled in Lattice Extreme Ablative Dose (LEAD) clinical trial [8].

## 2 Methods

### 2.1 Patients, MRI acquisition and manual ROIs delineation

Prostate cancer patients received treatment as part of the LEAD clinical trial (ClinicalTrials.gov: NCT01411319) at the University of Miami. The LEAD technique was described in detail by Pollack *et al* [8]. The trial was approved by the Institutional Review Board. Written informed consent was obtained from all patients. mpMRI scans were acquired using 3 T magnets (image acquisition parameters by vendor and type are summarized in Supplementary Table 1).

**Table 1:** Repeatability of the mean T2w between initial (baseline) and planning MRI in prostate, peripheral zone (PZ) and GTV based on Pearson r* and repeatability coefficient (*RC*).

Pre-RT		Before registration	After registration	
	ROI	manual	manual	automatic
Before normalization				
	Prostate	r = 0.72, RC = 894	r = 0.74, RC = 883	r = 0.76, RC = 854
	PZ	r *=* 0.75, RC *=* 1007	r = 0.78, RC = 964	r = 0.80, RC = 935
	GTV	r = 0.69, RC = 936	r = 0.70, RC = 912	r = 0.77, RC = 842
After normalization				
	Prostate	r = 0.62, RC = 157	r = 0.83, RC = 98	r **= 0.87, RC = 85**
	PZ	r = 0.53, RC = 300	r = 0.65, RC = 257	r **= 0.78, RC = 204**
	GTV	r = 0.36, RC = 270	r = 0.68, RC = 204	r **= 0.90, RC = 117**

The coefficients were calculated against the baseline intensities in the manual masks. The manual ROIs in the planning MRI were examined in their original space before registration (1st column) and after registration (2nd column) and were both compared with the automatic ROIs obtained from the baseline MRI (3rd column).

* All correlations were significant (*p* < 0.001).

**Fig. 1:**
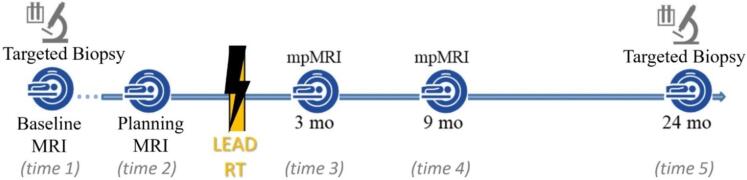
Timeline of mpMRI and biopsy acquisition. The baseline MRI was followed by MRI-Ultrasound (MRI-US) fused biopsy and fiducial placement. At the time of the planning CT, a limited fast MRI exam (no DCE-MRI) was carried out to acquire post-fiducial MRI data under the same conditions as the CT. After RT delivery, three post-RT scans were acquired at 3-, 9- and 24-months. An MRI-US biopsy was conducted at 24 months.

Fig. 1 illustrates the timeline of longitudinal mpMRI and biopsy acquisitions [2], [9]. The delineation procedure of ROIs (prostate, PZ, and GTV) was carried out in MIM (MIM Software Inc., Cleveland, OH) as described in Supplement, Appendix A. The manual ROIs in the baseline exams were used as input to *REHARM*, while the manual ROIs in subsequent exams were used as reference for the evaluation of *REHARM*’s registration method.

### 2.2 Image registration

The longitudinal morphological imaging changes were modeled using intra-subject deformable image registration between the initial T2w (time 1, reference) and follow-up T2w including the treatment planning (time 2) and post-RT MRIs (time 3, 4, 5) (Fig. 1). Overall, changes may be manifested due to global motion, soft tissue deformations (e.g. due to differences in the volume of bladder, bowel movement, biopsy and fiducial placement) and potential treatment-related shrinkage of lesions and prostate. The spatial transformation was estimated by first applying a linear mapping on T2w to account for global translation and rotation differences, and then a multi-scale deformable registration scheme using the *Demons* algorithm [10]. Specifically, we implemented a multi-scale registration scheme (with *shrink_factors* =[4,2] and *smoothing_sigmas* =[8,4]) and utilized in each scale the *FastSymmetricForcesDemonsRegistrationFilter* [11], which was developed to handle diffeomorphic transformations and to allow invertibility of the deformation (from time-backward to time-forward). While this algorithm is overall robust and efficient, it may fail in the RT targeted region due to intense signal changes caused by radiation-induced tissue alterations. To further boost registration accuracy, a second algorithm (the *ElastixImageFilter*^1^ class of *SimpleITK* in Python [12]) was utilized to refine the mapping by maximizing the mutual information between the images in the region within and around the prostate, while enforcing increased smoothness constraints. The smoothness constraints help to increase the regularization (uniformity) of the transformation and to provide a computable subspace, being necessary for uniqueness of the solution. The two deformation fields were concatenated by composition^2^ in order to obtain the complete longitudinal spatial transformation that maps each follow-up T2w in the reference space. According to this process, the masks delineated in the first timepoint were considered as automatic contours for the subsequent timepoints. Those automatic contours were evaluated by comparison with the corresponding ‘manual’ masks warped in the reference space.

### 2.3 Image intensity harmonization

The image harmonization method is an extension of our recently published work [13]. Briefly, intensity normalization was carried out in several steps: *(i)* applying a dual-stage MASK R-CNN classifier [14] trained for automatic segmentation of three (reference) structures on the T2w from male pelvis: gluteus maximus muscle (GM), femur and bladder; *(ii)* fitting a spline function between intensities averaged in these structures and pre-defined reference values; and *(iii)* using the spline function to scale the intensity of each pixel in T2w [13].

In *REHARM*, several improvements are proposed. Firstly, the segmentation of the reference structures was refined by three-dimensional (3D) shape post-processing. This helped to alleviate discontinuities caused by stacking the individual axial (2D) slices segmented by MASK R-CNN into a volumetric shape. The postprocessing included imputation of missing slices and 3D shape smoothing, as described in detail in Supplement, Appendix B.

Secondly, the intensity transformation was improved by introducing smoothness and monotonicity constraints on the signal. Specifically, after segmentation of GM, femur and bladder, the mean average intensities in those structures were used as “anchor” points to calculate a fitting function that maps those points to pre-assigned reference values (330, 660, and 990 a.u. (arbitrary units) for GM, femur and bladder, respectively). The fit was impeded when the three points were too close to each other or were inverted relative to the reference values. In *REHARM*’s implementation two boundary anchor points were added at 0 a.u. (minimum) and 1500 a.u. (maximum) intensity to constrain the intensity transformation within a standard scale. The anchor points were first piecewise-linearly interpolated to obtain a dense sampling of all intensities in the T2w image, and then introduced in a p-spline function to obtain a smooth, monotonic, non-negative mapping [15] (Supplement, Appendix C).

### 2.4 Evaluation

The registration accuracy was evaluated by calculating the DICE coefficient (*DSC*) [16] between the manually delineated ROIs in time 1 (ground-truth) and the deformably registered ROIs (from time 2, 3, 4 to time 1). The proposed normalization was compared to a common technique for image normalization, the histogram equalization [17]. Both methods were assessed by calculating the intersection of intensity histograms ($M_{\mathrm{inter}}$) between each patient to every other patient (Supplement, Appendix D) for T2w intensities within the prostate, PZ and GTV. To assess the longitudinal consistency of the framework, Pearson correlation and Bland-Altman analysis was performed between the mean T2w intensity in the examined ROIs in the two pre-RT scans. The repeatability coefficient was calculated as $\mathrm{RC}=\sqrt{2}∙1.96∙s_{w}$, where $s_{w}$ is the within-subject standard deviation. *RC* is related to the 95% limits of agreement that contain 95% of differences between repeated measurements on same subjects [18]. The smaller the *RC* values, the higher the repeatability in the T2w intensity estimation under the different settings.

## 3 Results

In the LEAD trial longitudinal imaging exams were carried out on 23 patients: 15 patients had 5 timepoints and 8 had 4 timepoints, resulting in a total of 107 T2w scans.

### 3.1 Image registration

**Fig. 2:**
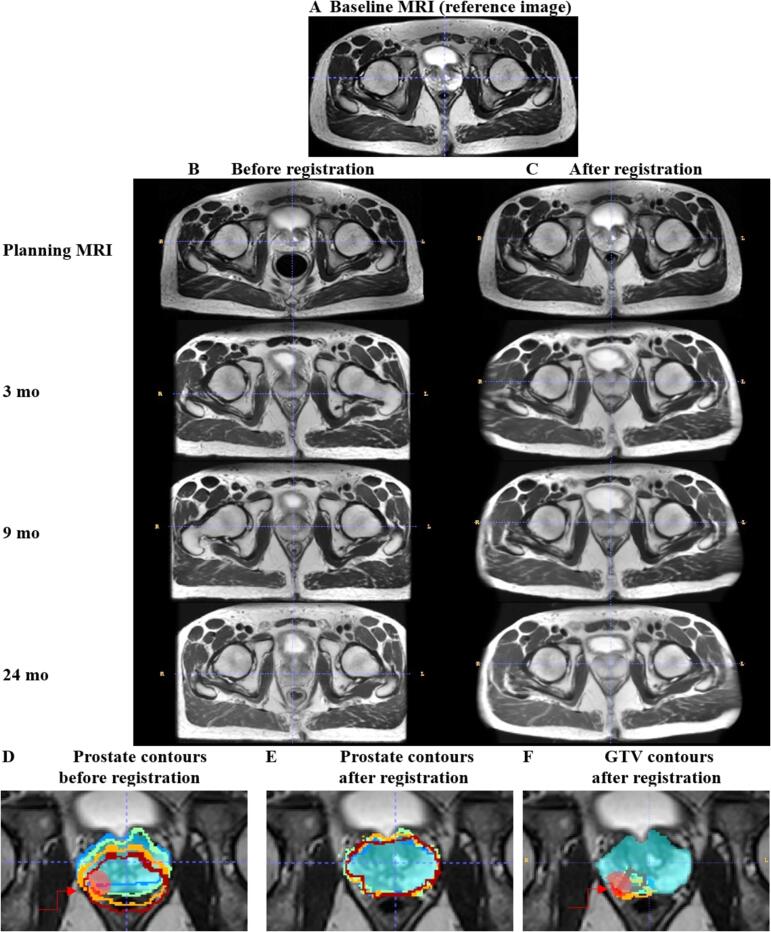
Registration of all timepoints to baseline T2w image (A). Rows 2–5: T2w images during planning MRI and post-RT before registration (B) and after registration (C) in baseline T2w space (A). All results before deformable registration shown in this figure have been produced using linear alignment (rotation and translation). Bottom row (D-F): The prostate (cyan volume) and GTV (red volume and red arrow) as outlined in baseline T2w are superimposed with corresponding prostate and GTV contours from planning MRI (blue), 3 mo (green), 9 mo (orange), 24 mo (red) post-RT before registration (D) and after deformable registration for prostate (E) and GTV (F). (For interpretation of the references to colour in this figure legend, the reader is referred to the web version of this article.)

The available follow-up T2w (including one pre-RT and three post-RT exams) were registered to the baseline MRI. The registration example in Fig. 2 demonstrates that simple linear alignment (Fig. 2**B)** is not sufficient to achieve good anatomical correspondence, as obtained after the deformable mapping (Fig. 2**C)**. For visual assessment of registration quality, Fig. 2 **(D–F)** illustrates also the overlap of the manual prostate and GTV contours of different timepoints before and after deformable registration in the baseline MRI space.

**Fig. 3:**
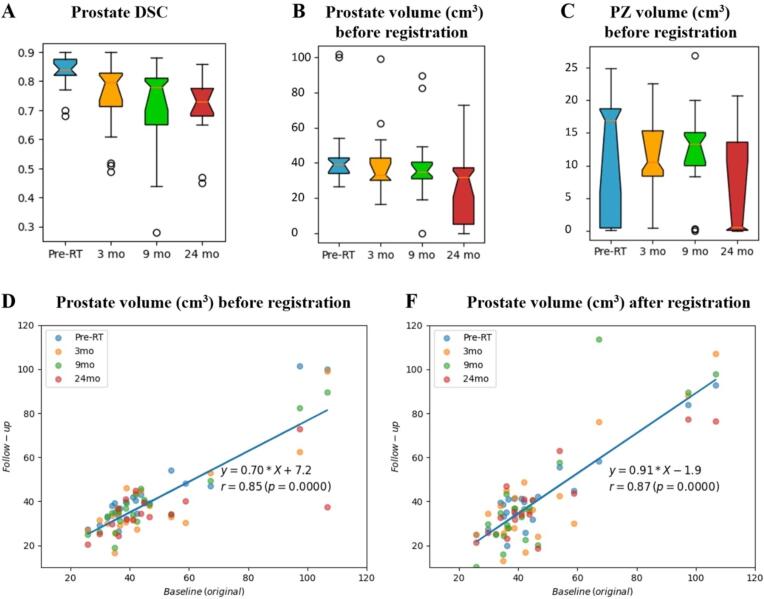
Evaluation of registration accuracy in the planning MRI (blue) and at 3 mo (orange), 9 mo (green), and 24 mo (red) post-RT MRIs. Top row: *DSC* between manual baseline and transformed prostate ROI (A); volume of prostate (B); volume of PZ (C). Bottom row: Scatter-plot of prostate volume with regression line between baseline ROIs (in x-axis) and follow-up ROIs (in y-axis) before registration (D) and after transformation in the space of baseline using *REHARM*’s deformable registration method (F). (For interpretation of the references to colour in this figure legend, the reader is referred to the web version of this article.)

The *DSC* between the manual and automatic masks is shown in Fig. 3**A**. One patient had double hip implants and the artifacts on T2w led to registration failure. This patient was excluded. It is evident that the registration accuracy is higher for the pre-RT scans (median ± stdev = 0.84 ± 0.06, followed by 0.80 ± 0.12, 0.78 ± 0.15, and 0.73 ± 0.11 for 3, 9, and 24 months post-RT, respectively) highlighting the effect of RT-induced and long-term changes on registration performance. The overall changes in prostate and PZ volumes are shown in Fig. 3**B, C**, indicating reduction in volumes shortly after RT (at 3 mo), especially for PZ. PZ volumes also show large variability, which is attributed to the highly variable glandular content in PZ between patients. Following RT, normal glands often undergo substantial shrinkage and deformation and, due to diverse gland content among patients, the effects of radiation therapy can vary significantly.

The overall Pearson correlation coefficient between the prostate volumes in manual mask at baseline and in deformably registered masks at subsequent timepoints was *r* = 0.87, *p* < 0.0001 (Fig. 3**F**). The slope of the regression line indicates overall 9% underestimation by the transformed volumes, significantly reducing the error of ∼ 30%, observed before registration (Fig. 3**D**).

### 3.2 Intensity harmonization

**Fig. 4:**
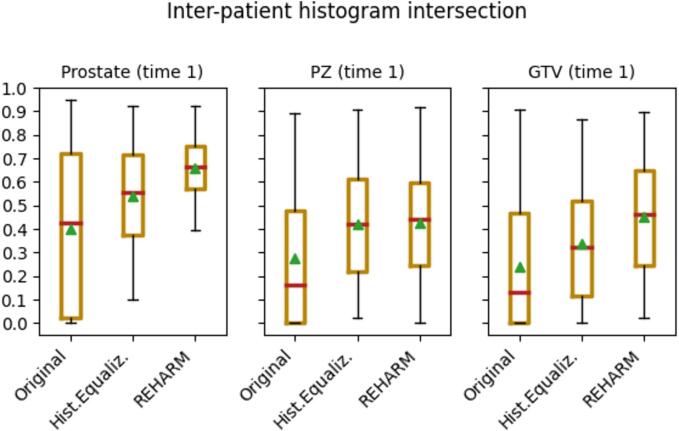
Inter-patient histogram intersections of the proposed framework (*REHARM*) compared to not normalized (original) and normalized images by histogram equalization (*Hist.Equaliz.*) for the whole prostate, peripheral zone (PZ) and GTV, respectively, in baseline MRI.

The inter-patient histogram intersection was computed between all combinations of two patients (*n* = 253 measurements for baseline MRI and *n* = 231 for planning MRI) in pre-RT images, before and after normalization with the *REHARM* methodology (Fig. 4). The $M_{\mathrm{inter}}$ values were extracted from the baseline MRI (time 1) and the co-registered planning MRI (time 2) within the prostate, PZ and GTV, contoured on baseline MRI. The procedure outperformed the histogram equalization approach as it resulted in higher histogram overlap in all examined regions at both time points (Fig. 4). The performance was similar in baseline and planning MRI, with the most profound improvement by *REHARM* in the GTV area (58% increase in median $M_{\mathrm{inter}}$ over histogram equalization, data not shown).

Furthermore, the repeatability of the T2w mean lesion intensity estimation in the two pre-RT scans was evaluated based on Pearson correlation and repeatability coefficient (*RC*) before and after *REHARM* normalization (Table 1). The normalized T2w intensities extracted from the automatic ROIs obtained by *REHARM* after registration markedly improved the longitudinal consistency metrics for all examined ROIs. All tests of the proposed image registration framework, with/without *REHARM* normalization, yielded a significant correlation between T2w average intensities in the two pre-RT scans. The largest *p*-value was 0.001 and the average 0.0002, thus *p*-values are not reported in the Table 1. For comparison, T2w normalization was also performed in the original image space, without co-registration. In this case, correlation dropped and became not significant for GTV (*r* = 0.36).

**Fig. 5:**
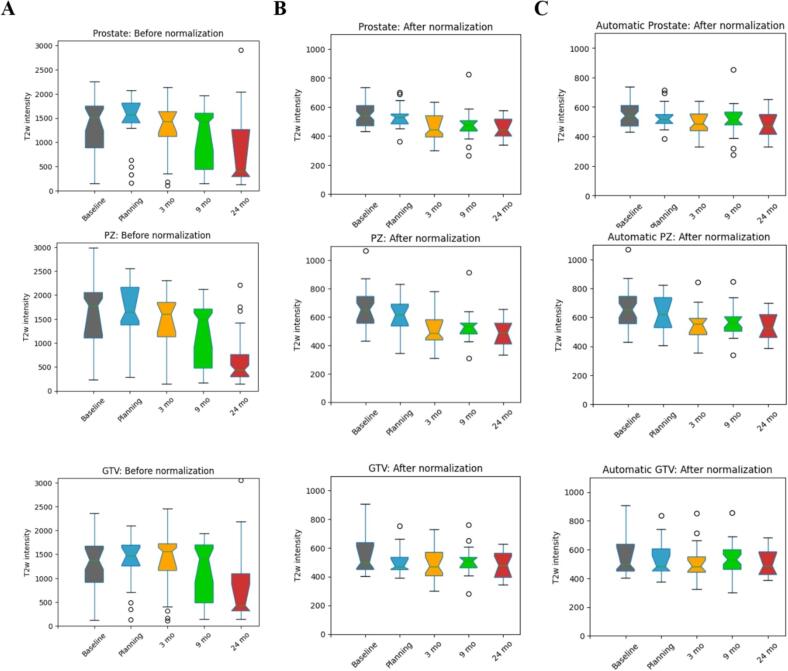
Changes in T2w intensity before and after RT. The box plots represent the mean T2w intensities in the prostate, PZ and GTV before (A) and after normalization using manual (B) and automatic (C) contours.

In the panels of Fig. 5 the ROIs’ longitudinal mean intensities are shown before and after normalization, using manual and automatic contours. After normalization: *(i)* the difference between pre- and post-RT distributions increased for prostate and PZ; *(ii)* the repeatability between the two pre-RT series improved; and *(iii)* the normalized intensities in manual and automatic contours were very similar, indicating the ability of *REHARM* to deliver quantitative features related to radiation-induced changes. Due to the reduction in the variation, interesting patterns of response of RT appeared. For prostate and PZ, a sharp decrease in the T2w intensity post-RT was observed, which was more pronounced for the PZ. The pre-RT (time 1, 2) T2w median intensities were averaged and compared with the post-RT values (average of time 3, 4, 5) revealing a 14% decrease in post-RT series for PZ, that was nearly double of the decrease observed for prostate (7.4%) (Fig. 5**C**). The T2w in the GTV following *REHARM* was almost constant (Fig. 5**C**), correcting for the drop of intensities at 24 mo observed before normalization (Fig. 5**A**, GTV). The statistical comparisons of the normalization effects are given in Supplement, Appendix E**.**

## 4 Discussion

An image analysis framework was developed to facilitate longitudinal follow-up of the prostate in MRI and automated assessment of prostate cancer response to treatment. While the T2w is the most utilized sequence in the MRI exam, there are no reports for quantitative evaluation of T2w pre-/post-RT. Relevant work includes MR image registration for prostate segmentation [19], [20] and for deformation-based planning, target volume definition and RT planning [21]. T2w normalization methods using reference tissues, such as femur head, levator ani muscle, pelvic bone, have also been proposed [22], [23]. Here we extended our recently published approach (Algohary *et al* [13]), where GM, femur and bladder were utilized as reference structures. This analysis uncovered several significant associations of T2w with tumor aggressiveness. Several other normalization techniques (histogram equalization [24], bladder scaling [25] and bladder centering [26]) failed to recover these associations.

*REHARM*’s image registration component enabled the automatic propagation of regions of interest in all timepoints. This is particularly challenging in the post-RT setting, where the prostate appears diffuse and hypo-isointense in T2w images due to glandular atrophy and fibrosis [27], [28]. The achieved median *DSC* of 0.84 between manual and *REHARM* contours on the planning MRI is approaching the level of interobserver variability (with a median *DSC* = 0.87) reported by Klein *et al* [29].

The GM and femur were utilized as reference structures for T2w intensity harmonization because they are located at a considerable distance from the RT fields. Similarly, the intensity of the bladder (T2w signal from urine) is not affected by radiation. Thus, the proposed normalization approach allows for investigation of longitudinal T2w changes post-RT. In Fig. 5, the difference between T2w (median) values of PZ and GTV increased from 20.1% (on original data) to 30.6% (following *REHARM*), demonstrating the increased discriminatory power of the normalized pre-RT T2w between the healthy and tumor tissues. On average, about 70% of the PZ is comprised mainly by prostate glands and the prostatic fluid in their lumina gives the rise of the T2 signal. The PZ showed great volumetric variability, related to the highly variable content of glandular tissue. We also observed variable “shrinking” of the PZ post-RT. The effect of the volume of healthy glands prior to RT and their role in response to RT is unknown. *REHARM* allows for systematic investigation of the patient’s specific response. Prostate tumors have a higher cellular density and are characterized by lower T2 intensities.

The observed T2w values decrease following RT in the PZ and to lesser extend in the prostate is consistent with the fact that RT is known to induce reduction and shrinking of the glandular tissue [30]. This is also confirmed in a recent report by Hanzlikova *et al* [31] where the T2 relaxation times also decrease following RT.

The two pre-RT MRI exams can be treated as repeat examinations, with the assumption that the placement of the fiducial markers and the limited biopsies conducted between the two exams do not have a significant impact on the T2w signal (Fig. 1). Assessment of the repeatability of T2w values in pre-RT and post-RT scans showed that the normalization, applied on coregistered images, improved repeatability in T2w values versus manual delineation of ROIs.

Contouring the prostate, PZ, and GTV in these longitudinal imaging series is a time-consuming process, particularly in the post-RT series due to the diminished contrast between tumor and normal appearing prostate tissue, as well as the overall changes in the prostate gland. The developed image registration and T2w normalization sets the groundwork for conducting radiomics and Δradiomics studies on a large scale. Analyzing radiomic feature trends observed in longitudinal imaging studies over the course of therapy holds great promise and has the potential to significantly enhance the prediction of treatment response models [2], [32].

There are several limitations. First, the validation of the methodology was performed on data from the same clinical trial. External validation on independent datasets is warranted. Because the study cohort was relatively small, the effect of androgen deprivation therapy administration on the procedure was not investigated. Also, because of the signal loss post-RT, the evaluated image similarity in the treated region is locally suboptimal.

Extension of *REHARM* for improved robustness includes the investigation of other deformable registration algorithms that we have implemented in the past [33], [34], [35], [36], which are generally slower than *Demons* but may improve performance.

In conclusion, the developed methodology allows to automate longitudinal analysis reducing data acquisition-related variation and improving consistency. The quantitative characterization of RT-induced changes in T2w will lead to new understanding of radiation effects enabling prediction modeling of RT response.

## Author contributions

EZ, RS, and AP contributed to the conception and design of the study. BS, SP, MA, AP, and AP conducted the clinical trials and provided contours of Gross Tumor Volumes (GTVs), patient clinical and imaging data for use in the study. PC reviewed imaging studies and provided PIRADS scoring. OK and SG reviewed and scored biopsy tissues. RS, and AB provided prostate contours and mapped GTVs on longitudinal imaging datasets. EZ, AA, AB, VW, and RS organized the patient database. EZ, AA, and RS designed the normalization procedure. AB developed the deep learning network for the normalization. EZ developed methodology and designed software for co-registration. EZ wrote the overall software for normalization, co-registration and visualization. PP and JF advised on the physical interpretation of the MRI sequences. RS supervised the overall project. EZ wrote the first draft of the manuscript and RS provided substantial review and editing. All authors contributed to manuscript revision, read, and approved the submitted version.

## Declaration of competing interest

The authors declare the following financial interests/personal relationships which may be considered as potential competing interests: MA declares consulting or advisory Role at Varian Medical Systems and research funding from Varian Medical Systems (Inst) and Varian Medical Systems (Inst). ADP declares consulting or advisory role at Merck and Pfizer and research funding from Veracyte (Inst). AP declares a past role as co-chair of the RTOG/NRG GU Translational Research Program.

## Footnotes

1 https://simpleitk.org/doxygen/v2_2/html/classitk_1_1simple_1_1ElastixImageFilter.html.

2 The composition of two deformation fields $D_{\mathrm{ij}}\left(x\right)$ and $D_{\mathrm{jk}}\left(x\right)$ that map voxel x from i to j and from j to k respectively, is $D_{\mathrm{jk}}\left(D_{\mathrm{ij}}\left(x\right)\right)$.
