# Supplementary material for: Integrated framework for quantitative T2-weighted MRI analysis following prostate cancer radiotherapy
Source: Phys Imaging Radiat Oncol. 2024 Oct 24;32:100660. doi: 10.1016/j.phro.2024.100660 (PMC11574798; doi:10.1016/j.phro.2024.100660)
Supplement: Supplementary Data 1 [file mmc1.docx]

**Supplementary Material**

**Supplementary Table 1** MRI acquisition parameters for axial T2-weighted MRI sequence.

| **Scanner** | **N** | **TE**  **Min - Max** | **ETL** | **TR**  **Min - Max** | **Matrix** | **Voxel Size**  **(mm)** |
| --- | --- | --- | --- | --- | --- | --- |
| **GE** | 81 | 81.58-124.74 | 16, 24 | 5070-10998 | 256×256×72 | 1.250×1.250×2.5 |
|  | 2 | 99.53-105.17 | 16 | 4882-4794 | 256×256×72 | 1.250×1.250×3.0 |
|  | 1 | 83.34 | 16 | 7132 | 256×256×95 | 1.406×1.406×2.5 |
| **Siemens** |  |  |  |  |  |  |
| **Skyra** | 11 | 114 | 21 | 6100 | 512×512×72 | 0.703×0.703×2.5 |
| **Avanto** | 1 | 88 | 27 | 4082 | 512×512×73 | 0.703×0.703×2.5 |
| **Sonata** | 2 | 94 | 23 | 4860 | 512×384×72 | 0.625×0.625×2.5 |
| **Symphony** | 2 | 89 | 23 | 5960 | 512×384×72 | 0.703×0.703×2.5 |
| **TrioTim** | 7 | 112, 89 | 21 | 6000 | 512×384×72 | 0.703×0.703×2.5 |

Abbreviations: TE=Echo Time, ETL = Echo Train Length, TR=Repetition Time

1. Delineation of Regions of Interest (ROIs)

The ROIs were contoured by imaging experts (RS, ALB) with more than 25 years combined expertise in prostate imaging and reviewed by radiation oncologists (MCA, AP) with extensive expertise in genitourinary malignancies. The prostate and Peripheral Zone (PZ) were manually contoured on the baseline MRI by inspecting the T2-weighted MRI (T2w) sequence, whereas the Gross Tumor Volumes (GTVs) were determined based on mpMRI-defined suspicious abnormalities using PI-RADSv2 [1]. The findings were also confirmed by heatmaps, generated by a habitat risk scoring (HRS) system described in Stoyanova *et al* [2]. Transferring of the ROIs onto subsequent scans was performed also manually in MIM by adjusting prostate and PZ contours to account for prostate size changes following RT, whereas the GTV was positioned over the area of the RT boost without changing the lesion volume. In this process, anatomical landmarks, such as the urethra or ejaculatory ducts, were used for visual guidance.

1. Imputation of missing 2D slices and 3D shape smoothing

The three reference structures obtained from the MASK R-CNN are refined based on interpolation between existing slices in the axial plane and 3D shape smoothing. For multifocal structures, i.e. femur and GM, the right and left segments are first automatically separated and then individually processed. The whole post-processing procedure is described next.

1. For each mask *M*, discrimination of right-left (R-L) structures is performed using unsupervised clustering by *Kmeans* (with *k=*2) on the coordinates of the voxels inside the whole mask. If $\left( x,y,z \right)$ indicate the coordinates of the mask in R-L, anterior-posterior and inferior-superior axes, then two cluster centroids are used to initialize the *Kmeans* algorithm on the two extremes in the R-L axis and on the average in the other two axes:

$$\left( x_{1},y_{1},z_{1} \right)=\left( \min_{i\in M} x_{i},\frac{1}{\left| M \right|}\sum_{i\in M} y_{i},\frac{1}{\left| M \right|}\sum_{i\in M} z_{i} \right)$$

$$\left( x_{2},y_{2},z_{2} \right)=\left( \max_{i\in M} x_{i},\frac{1}{\left| M \right|}\sum_{i\in M} y_{i},\frac{1}{\left| M \right|}\sum_{i\in M} z_{i} \right)$$

where $min$, $max$ indicate the minimum and maximum values, respectively, $i$ the index on voxels and $\left| M \right|$ the volume (=number of voxels) of mask *M*. *Kmeans* then partitions the voxels into two sets (clusters) such that the within-cluster variances are minimized. Due to larger voxel-to-voxel distance across the R-L axis than across the other two axes, and the selected centroid initialization, the algorithm leads to right-left partition.

1. For each structure, a 2D slice at *z* (coordinate across the inferior-superior axis) is considered missing if slices exist at coordinates smaller and larger than *z*. For each missing slice, the lower and upper nearest existing frames are identified and used to estimate the motion (shift) between them.
2. The center of mass in each frame is calculated and the shift between the two slices is estimated using splines of order 3. The motion between the two nearest frames is interpolated by shifting frame 1 towards frame 2 and inversely.
3. Then the weighted average of the forward and backward shift is calculated, using as weight the relative distance from each frame.
4. The obtained gray-scale image is thresholded to binarize the final 3D mask.

After slice imputation across the axial direction, 3D shape (boundary) smoothing is performed by applying

- 1. intensity-based smoothing using recursive Gaussian filtering and intensity thresholding and
  2. binary morphological closing to remove small holes and tube-like structures in the interior or at the boundaries of the 3D masks.

An example of the application of the MASK R-CNN model for segmentation of axial 2D slices is shown in **Fig. S1** top row. Postprocessing of the obtained volumetric masks allows to first identify left-right structures and impute missing slices for each of them (**Fig. S1**, middle row), and then improve the 3D shape characteristics (**Fig. S1**, bottom row). As a note, the impact of the segmentation accuracy on T2w normalization is not significant, because suboptimally segmented parts are rare and relatively small. Also, since the T2w normalization algorithm is based only on average intensities from each reference structure, the effect of random outliers is averaged out, making the algorithm less sensitive to segmentation inaccuracy.


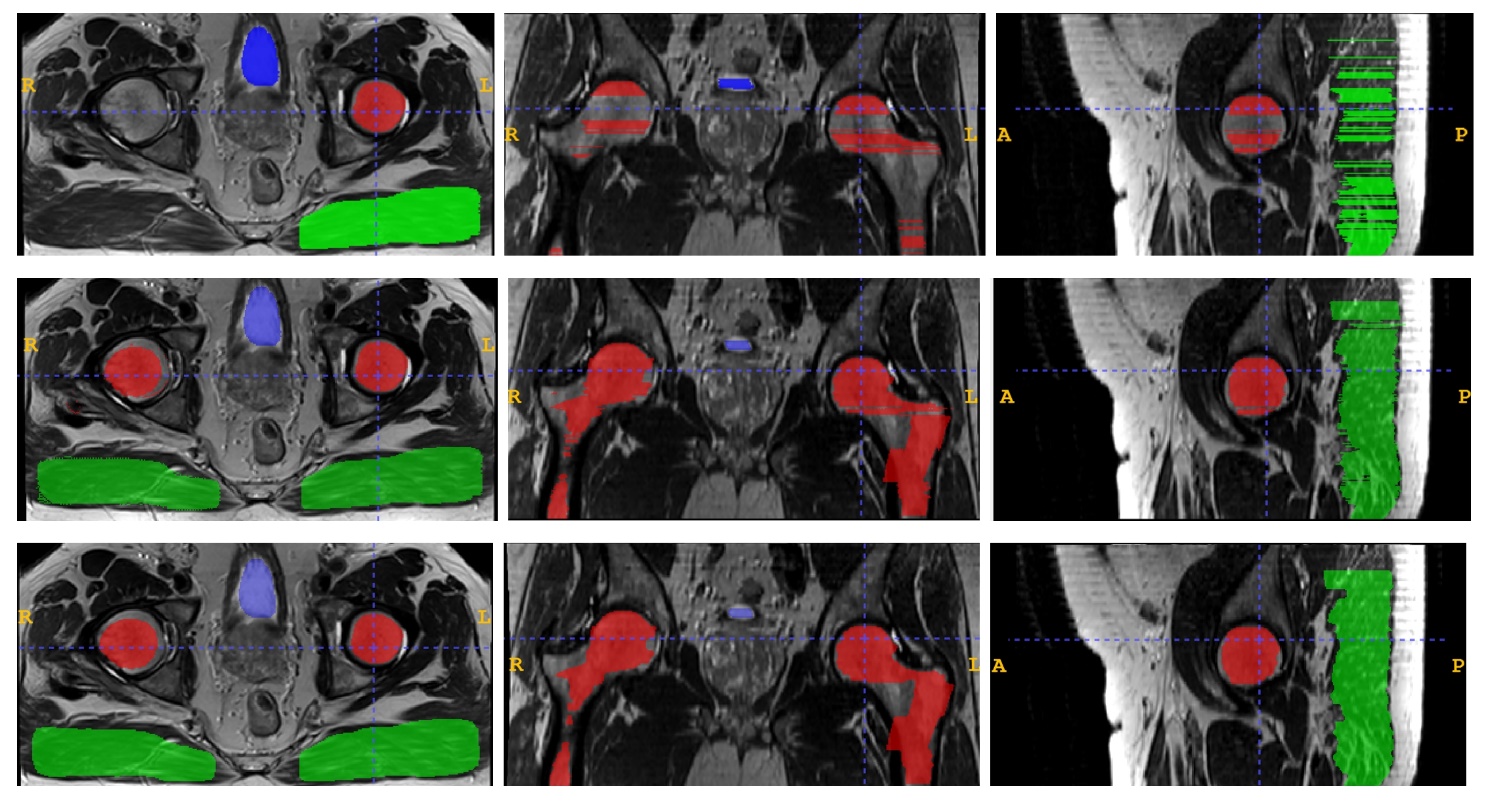


**Fig. S1.** T2w image segmentation and postprocessing of reference tissue masks. The columns left to right illustrate an axial, coronal and sagittal T2w image overlaid with segmentation masks: GM (green), femur (red), bladder (blue). The rows show the volumetric masks as obtained by the 2D MaskRCNN network (top), after imputation across axial slices (middle), and after 3D shape smoothing (bottom).

1. Fitting function mapping T2w intensities to reference values

Given a set a T2w intensity reference values at predefined anchor points, a fitting function is calculated and used to transform the total range of T2w intensity values. First piecewise linear interpolation between the anchor points is performed to obtain a dense sampling of all intensities in the T2w image and then a p-spline function is fitted on the dense points for unimodal (showing only one peak) smoothing [3, 4], helping to eliminate abrupt changes of slope in the interpolation function around the anchor points. In addition, non-negativity and monotonicity of the spline function is enforced to reduce the effect of potential reference tissue segmentation inaccuracies. This helped to increase robustness of the intensity normalization process, especially in the very rare case of reference tissue segmentation errors that led to a reverse order in the estimated reference tissue values. The **Fig. S2** shows an example of previous [4] and proposed intensity transformation functions. Fitting of a single spline function on the intensities of the three reference tissues when two of them have similar values, may cause non-monotonic mapping (**Fig. S2**, left, ‘original fit’), such that some tissues with original intensities higher than GM (and less than femur) get transformed to intensities lower than the reference GM value.


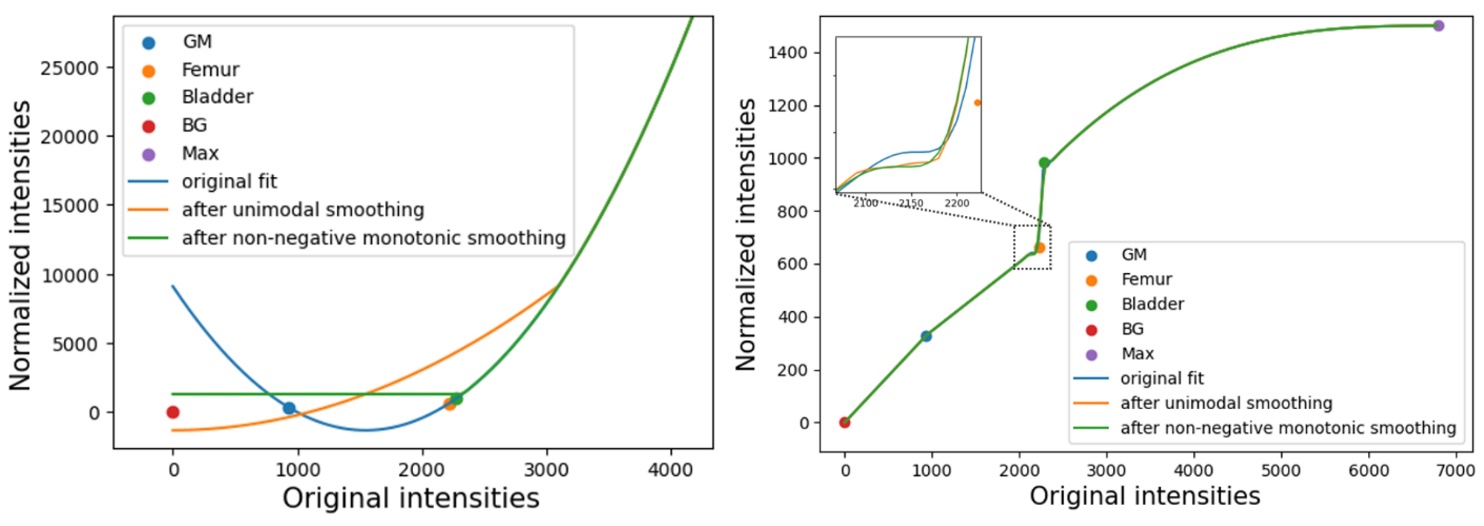


**Fig. S2.** Example of intensity mapping functions for unconstrained (three anchor points) single-fit (left) and constrained (five anchor points) piece-wise fit (right). In both case the transformation function is shown before smoothing (original fit, blue line) and following unimodal and non-negative smoothing (orange and green line, resp). Left: The mean T2w values in the three reference tissue (GM, Femur and Bladder) are mapped to three reference values (330, 660, and 990 a.u.), resp). Right: Spline fit after interpolation between five anchor points that include background (BG) and Max. In both cases unimodal and non-negative smoothing improve the transformation function, but the improvement is more profound in the unconstrained fit (left), whereas for the constrained fit (right)it is only marginal (as shown in zoomed region 2100-2200). GM: gluteus maximus muscle; BG: background intensity (0), Max: maximum normalized intensity (1500).

This can be overcome with the new multi-step transformation procedure that performs monotonic smoothing. Moreover, the lack of standard limits (**Fig. S2**, left) might increase the background/noise intensity and cause unlimited growth of maximum intensity values. This is addressed by adding two more anchor points to standardize the background (BG) and maximum (Max) intensity values (**Fig. S2**, right).

1. Inter-patient histogram intersection

If $H_{x}$ and $H_{y}$ represent the image intensity histograms (normalized to the total number of voxels) of patient *x* and patient *y*, respectively, and *b* represents the number of histogram bins (set to 100), then the histogram intersection is calculated as

$$M_{inter}\left( H_{x},H_{y} \right)=\sum_{i=1}^{b} min(H_{x}(i),H_{y}(i))$$

The $M_{inter}$ values range between 0 and 1, with higher values indicating greater overlap of intensity distribution between different patients.

1. Inter-patient histogram intersection

In order to quantitatively assess the effect of normalization in distinguishing RT-induced changes in registered post-RT images, pre-RT and post-RT intensities (**Figure 5** of main manuscript) were compared using paired samples t-test (**Supplementary Table 2**). Before normalization, while the T2w values decreased after RT, the differences were not significant due to large intensity variation, with the exception of peripheral zone (PZ) at 9 and 12 mo. However, after normalization, all comparisons were statistically significant (*p* < 0.05) for PZ and prostate (**Figure 5B, C** of main manuscript). The majority of comparisons for the GTV were not significant, confirming the lack of detected changes in T2w intensities in the GTV.

| **Supplementary Table 2** *p*-values of intra-patient paired *t*-tests between pre-RT (average of baseline and planning MRI) and post-RT samples |
| --- |
| \|  \|  \| manual \| \| \|  \| automatic \| \| \| \| --- \| --- \| --- \| --- \| --- \| --- \| --- \| --- \| --- \| \|  \|  \| 3 mo \| 9 mo \| 24 mo \|  \| 3 mo \| 9 mo \| 24 mo \| \| *p*-values before normalization (A) (corresponding distributions not shown) \| \| \| \| \| \| \| \| \| \| Prostate \|  \| 0.289 \| 0.068 \| 0.066 \|  \| 0.491 \| 0.131 \| 0.059 \| \| PZ \|  \| 0.153 \| 0.034 \| 0.010 \|  \| 0.281 \| 0.069 \| 0.047 \| \| GTV \|  \| 0.577 \| 0.186 \| 0.100 \|  \| 0.691 \| 0.309 \| 0.104 \| \| *p*-values after normalization (B) (C) \| \| \| \| \| \| \| \| \| \| Prostate \|  \| 0.000 \| 0.000 \| 0.000 \|  \| 0.002 \| 0.035 \| 0.000 \| \| PZ \|  \| 0.000 \| 0.000 \| 0.000 \|  \| 0.000 \| 0.005 \| 0.000 \| \| GTV \|  \| 0.050 \| 0.080 \| 0.010 \|  \| 0.146 \| 0.312 \| 0.008 \| |

**References**

[1] A. R. Padhani, J. Weinreb, A. B. Rosenkrantz, G. Villeirs, B. Turkbey, J. Barentsz, Prostate Imaging-Reporting and Data System Steering Committee: PI-RADS v2 Status Update and Future Directions *Eur Urol,* 2018, <http://www.ncbi.nlm.nih.gov/pubmed/29908876>.

[2] R. Stoyanova, F. Chinea, D. Kwon, I. M. Reis, Y. Tschudi, N. A. Parra *et al.*, An Automated Multiparametric MRI Quantitative Imaging Prostate Habitat Risk Scoring System for Defining External Beam Radiation Therapy Boost Volumes *Int J Radiat Oncol Biol Phys,* 102, 2018, 821-829, <https://www.ncbi.nlm.nih.gov/pubmed/29908220>.

[3] P. H. Eilers, Unimodal smoothing *Journal of Chemometrics: A Journal of the Chemometrics Society,* 19, 2005, 317-328.

[4] A. Algohary, M. Alhusseini, A. L. Breto, D. Kwon, I. R. Xu, S. M. Gaston *et al.*, Longitudinal Changes and Predictive Value of Multiparametric MRI Features for Prostate Cancer Patients Treated with MRI-Guided Lattice Extreme Ablative Dose (LEAD) Boost Radiotherapy *Cancers,* 14, 2022, <Go to ISI>://WOS:000858518000001.
